# Sympathetic Neural Mechanisms Underlying Attended and Unattended Blood Pressure Measurement

**DOI:** 10.1161/HYPERTENSIONAHA.121.17657

**Published:** 2021-08-09

**Authors:** Guido Grassi, Fosca Quarti-Trevano, Gino Seravalle, Raffaella Dell’Oro, Jennifer Vanoli, Gianluca Perseghin, Giuseppe Mancia

**Affiliations:** Clinica Medica, Department of Medicine and Surgery, University of Milano-Bicocca (G.G., F.Q.-T., G.S., R.D., J.V.).; Department of Medicine and Rehabilitation, Policlinico di Monza, Monza, Italy (G.P.).; Policlinico di Monza, Monza and University Milano-Bicocca, Milan, Italy (G.P., G.M.).

**Keywords:** blood pressure, heart rate, hypertension, muscle, sympathetic nervous system

## Abstract

Whether blood pressure (BP) values differ when BP is measured with or without the presence of a doctor (attended and unattended BP measurements) is controversial, and no information exists on whether and to what extent neurogenic mechanisms participate at the possible BP differences between these measurements. In this study, we assessed continuous beat-to-beat finger systolic BP and diastolic BP, heart rate, muscle, and skin sympathetic nerve traffic (microneurography) before and during BP measurement by an automatic device in the presence or absence of a doctor. This was done in 18 untreated mild-to-moderate essential hypertensive patients (age, 40.2±2.8 years, mean±SEM). During attended BP measurement, there was an increase in systolic BP, diastolic BP, heart rate, and skin sympathetic nerve traffic and a muscle sympathetic nerve traffic decrease, the peak changes being +5.3%,+8.4%,+9.4%,+30.9%, and −15.2%, respectively (*P*<0.05 for all). In contrast, during unattended BP measurement, systolic BP, diastolic BP, heart rate, and skin sympathetic nerve traffic were modestly, albeit in most instances significantly, reduced, whereas muscle sympathetic nerve traffic remained almost unchanged. During unattended BP measurement, peak systolic BP was 14.1 mm Hg lower, peak heart rate was 10.6 bpm lower, and peak skin sympathetic nerve traffic was 8.5 bursts/min lower than the peak values detected during attended BP measurement. Thus the cardiovascular and neural sympathetic responses to the alerting reaction elicited by BP measurement in the presence of a doctor are almost absent during unattended BP measurement, during which, if anything, a modest cardiovascular sympathoinhibition occurs. This has important implications for comparison of studies using these different BP measurement approaches as well as for decision concerning threshold and target BP values for treatment.

Following its use in the SPRINT (Systolic Blood Pressure Intervention Trial),^1^ unattended blood pressure (BP) measurement, that is, self-measurement of BP when patient is left alone in the room, has been the object of attention and controversy by the cardiovascular research community. A controversial aspect is whether this type of BP measurement provides values that are lower than those associated with traditional measurements, that is, those obtained in the presence of medical personnel. Although denied by the SPRINT investigators,^2^ this is supported by the results of studies which have shown (1) attended BP measurement to be associated with an increase in BP,^3–6^ which, in contrast, does not appear to change if BP is measured automatically or semiautomatically when the patient is alone in the room^4–6^ and (2) unattended BP measurement to be lower than traditionally measured office BP, although variably modestly to markedly different values have been reported in different studies.^4–10^ This is related to the clinical problem of which BP may provide a better insight on patients’ usual BP values,^8–10^ leading to a better diagnosis of hypertension and a more accurate prediction of the risk of future cardiovascular events at the individual patient level, two issues on which evidence is limited.^8,9^

**See Editorial, pp 1134–1137**

No study has ever assessed beat-to-beat BP and heart rate (HR) during unattended BP measurement, thereby providing direct evidence on whether and to what extent this procedure is associated with BP and HR changes. This has been the goal of the present study in which unattended BP measurement was used in hypertensive patients under continuous beat-to-beat BP and HR recording. Continuous beat-to-beat recording was extended to efferent postganglionic sympathetic nerve traffic to skin and skeletal muscle districts via the microneurographic technique^11^ to obtain information also on the neural involvement of the observed hemodynamic changes. Data were compared with those obtained in the same patients during the attended BP measurement procedure, which has been previously shown to be characterized by a BP rise, a tachycardia, and a sympathetic overdrive.^3,11,12^

## Methods

The data that support the findings of this study are available from the corresponding author on reasonable request.

### Population

The study population consisted of 28 patients with a newly diagnosed mild-to-moderate high BP state recruited for this specific study between 2018 and 2020. However, due to loss or instability of sympathetic nerve traffic recordings (see below), the study was successfully completed in 18 patients (14 males, 4 females, age 40.2±2.8 years, [means±SEM]) with an uncomplicated essential hypertension of mild-to-moderate degree. Patients were not considered for the present study if they exhibited (1) BP values outside the range defining a moderate hypertensive state; (2) clinical, laboratory, or instrumental evidence of secondary causes of the BP elevation; (3) signs, symptoms, or history of heart failure, coronary, or cerebrovascular events; (4) alterations in echocardiographically determined left ventricular diameters, left ventricular ejection fraction or left ventricular mass; (5) atrial fibrillation or other major cardiac arrhythmias; (6) clinical or instrumental evidence of valvular heart disease; (7) history of excessive alcohol consumption (>60 g/d in men and >40 g/d in women), or use of antidepressant drugs; (8) major concomitant diseases, such as renal insufficiency, diabetes, obesity, severe sleep apnea, and other conditions known to affect the neuroadrenergic function.^13^ All patients were pharmacologically untreated and their evaluation was carried out on an outpatient basis. The study protocol was approved by the Ethics Committees of the Institutions involved. All patients gave written consent to the study after being informed of its nature and purpose.

### Measurements

BP was measured by a validated automated oscillometric device (Omron HEM-9210T; Omron Healthcare, Co, Ltd, Kyoto, Japan),^14^ which was programmed to automatically perform BP measurements. It was also measured continuously by a finger photoplethysmographic device (Finapres 2300; Engle-wood, CO) capable of proving accurate and reproducible beta-to-beat systolic BP (SBP) and diastolic BP (DBP) values.^11,12^ HR was monitored continuously by the R wave of an electrocardiographic lead. Respiration rate was monitored by a strain gauge pneumograph positioned at the midchest level. Multiunit recordings of efferent postganglionic sympathetic nerve activity to the skeletal muscle (muscle sympathetic nerve traffic [MSNA]) or the skin (skin sympathetic nerve traffic [SSNA]) district were obtained through a tungsten microelectrode inserted into the right or left peroneal nerve posterior to the fibular head, as previously described.^11–13^ Simultaneous MSNA or SSNA, beat-to-beat BP, and beat-to-beat HR recordings were digitized with a sampling frequency of 1000 Hz (PowerLab Recording System Model ML870 8/30; AD Instruments, Bella Vista, New South Wales, Australia). The muscle or skin nature of the neurograms was assessed by the criteria outlined in previous studies.^11–13^ Neurograms were accepted only if they did not show simultaneous skin and muscle sympathetic nerve traffic and if the signal-to-noise ratio was >3.^11–13^ MSNA and SSNA were quantified over each minute as number of bursts. This quantification provides values that have been shown to be highly reproducible on a short-term basis when assessed twice in the same session by a single investigator.^15^

### Protocol and Data Analysis

**Figure 1.**
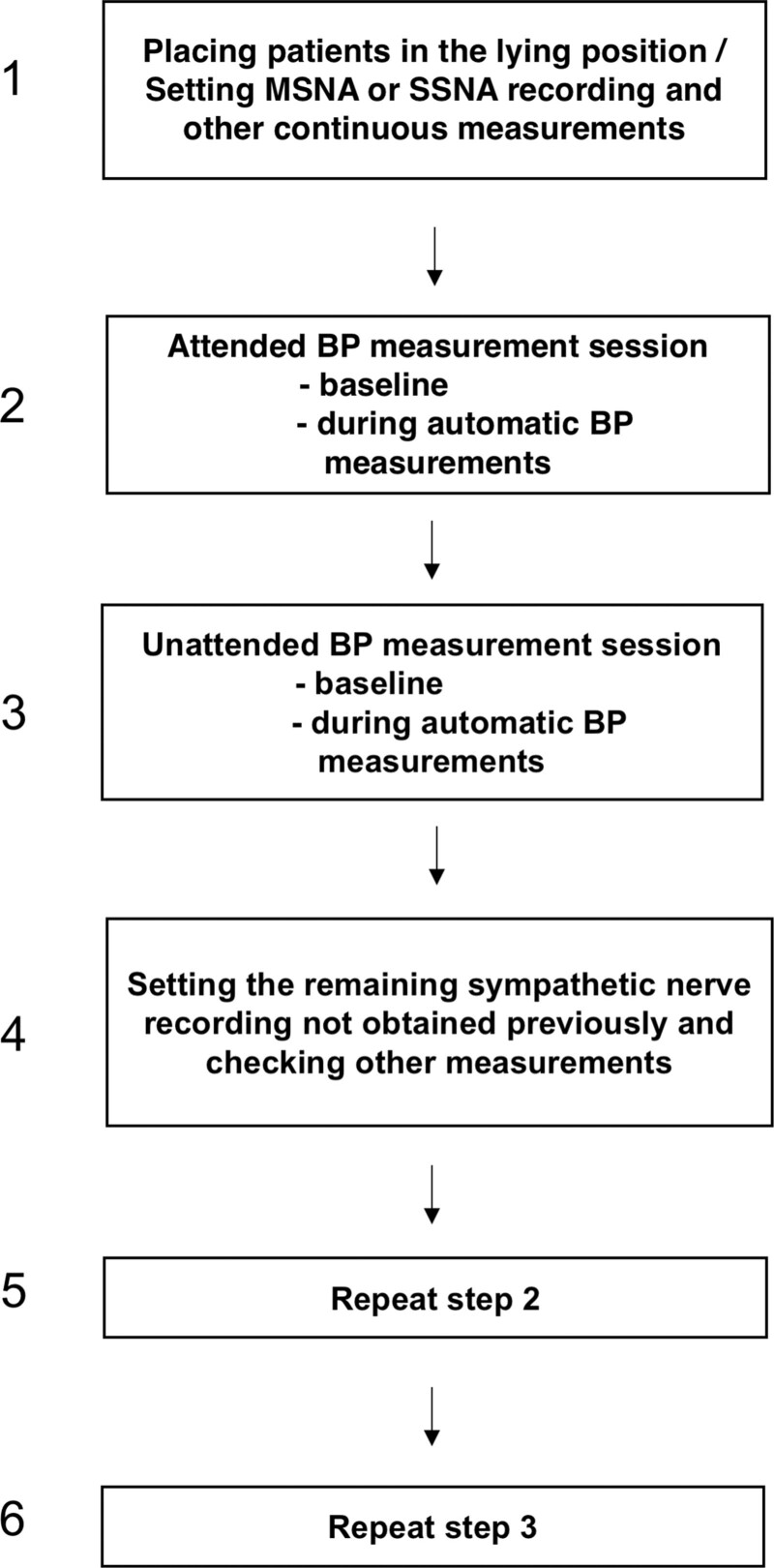
**Schematic drawing of the study design.** The sequence of unattended/attended session was variable between patients. BP indicates blood pressure; MSNA, muscle sympathetic nerve traffic; and SSNA, skin sympathetic nerve traffic.

All 18 patients were evaluated 1 to 2 weeks after a third medical visit when the mild-to-moderate essential hypertensive state had been conclusively established. As shown in Figure 1 the protocol of the study was as follows: (1) the patient was asked to come to the outpatient clinic in the afternoon, brought to the laboratory, placed in the lying position, and fitted with the various measuring devices, (2) following its insertion in the peroneal nerve, the microelectrode was manipulated by the investigator until MSNA or SSNA was detected. The neural and the other recordings were then started and continued throughout the study steps, (3) in the presence of a doctor data were collected during an initial 10-minute baseline period and during a second 10-minute period, in the second part of which the automatic device measured BP at 1-minute intervals from each other, (4) data were collected as in step 3 with the patient alone in the room, (5) the microelectrode was repositioned to detect the sympathetic nerve traffic not obtained in the previous recording (SSNA or MSNA), and (6) data collection was repeated as in steps 3 and 4. In 10 patients, the unattended recording steps preceded the attended ones, whereas the reverse was the case in the remaining patients. Data were analyzed by a single investigator unaware of the belonging of the measurements to the attended or unattended recording period. For each variable, mean values were obtained for each 1-minute recording. The peak minute value during the attended or unattended automatic BP measurement was compared with the average value of the baseline period. Values from individual patients were averaged for the group and differences between mean values were assessed by 2-way ANOVA for repeated measurements. Student *t* test for paired observations was used to locate the statistical significance of the difference, after the Bonferroni correction. The Pearson correlation coefficient was used to determine the relationships between MSNA, SSNA, HR, SBP, or DBP. All analyses were performed with SAS software version 9.3 (SAS Institute Inc, Cary, NC). The symbol ± refers to the SEM. A *P*<0.05 was taken as the level of statistical significance.

## Results

**Figure 2.**
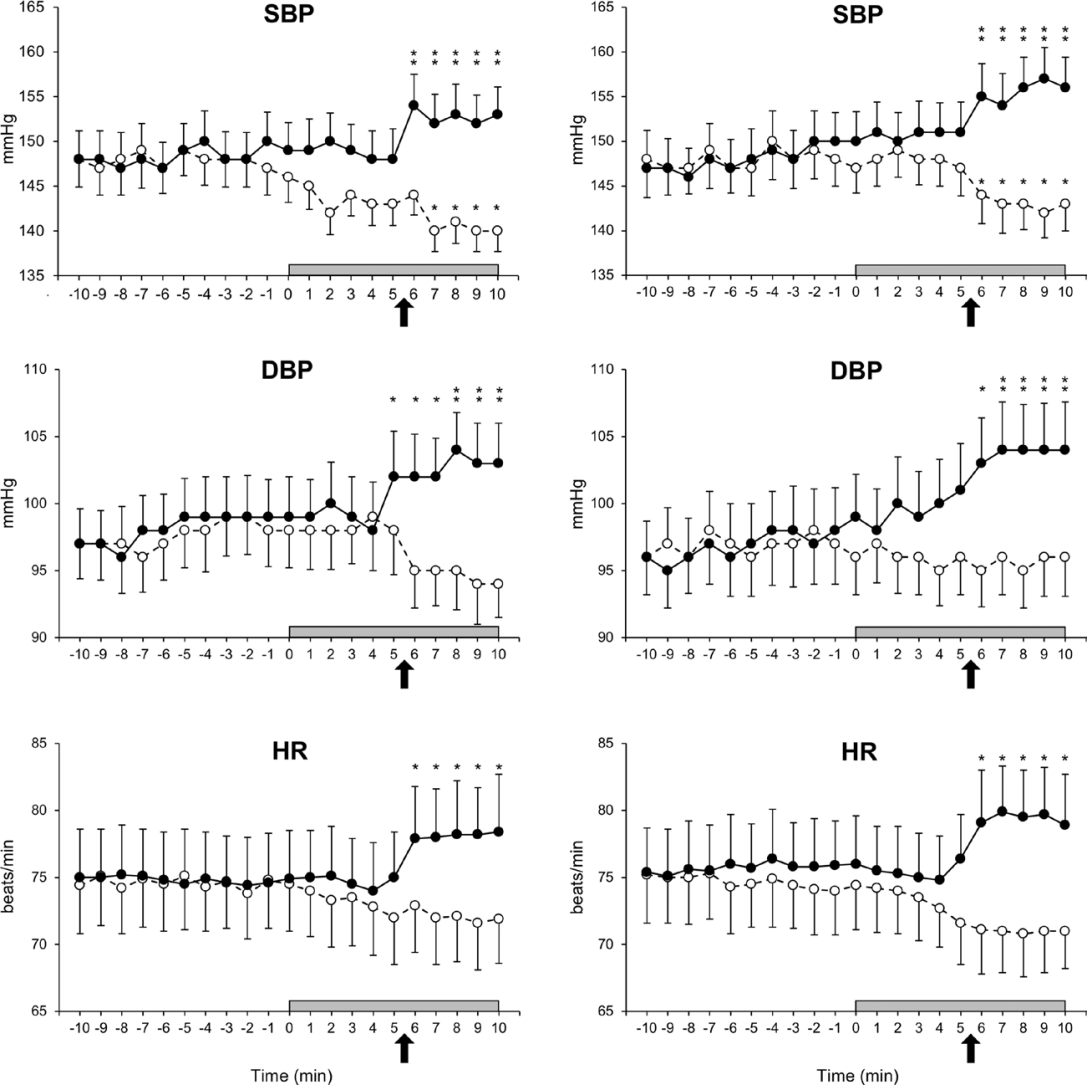
**Beat-to-beat finger systolic blood pressure (SBP), diastolic blood pressure (DBP), and heart rate (HR) values recorded before and during unattended (dashed lines, open circles) and attended (continuous lines, closed circles) automatic blood pressure measurement session. Left** parts refer to the session during which muscle sympathetic nerve traffic was recorded during the unattended and attended BP measurement, whereas the **right** ones to the session during which skin sympathetic nerve traffic was recorded during unattended or attended BP measurement. The arrows indicate the time in each session during which automated BP measurement started in presence or absence of the doctor. Data are shown as means±SEM. Asterisks (*P<0.04, **P<0.01) refer to the level of statistical significance between values obtained during unattended or attended BP measurement and baseline values.

**Figure 3.**
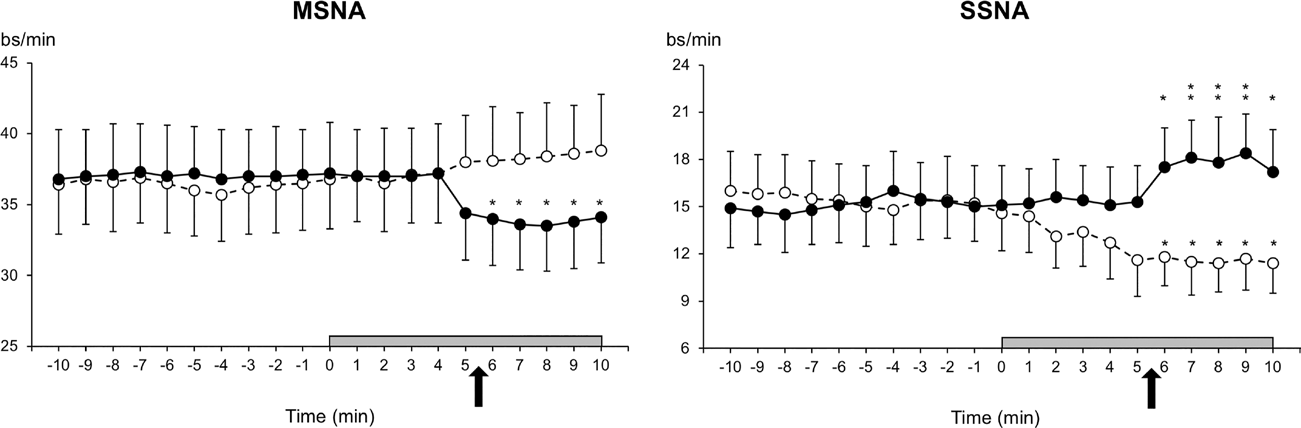
**Muscle sympathetic nerve traffic (MSNA), expressed as bursts frequency over time (bursts/min) and skin sympathetic nerve traffic (SSNA), expressed as bursts frequency over time (bursts/min), recorded before and during the unattended (dashed lines, open circles) and attended (continuous lines, closed circles) automatic blood pressure measurement session.** The arrows indicate the time in each session during which automated BP measurement started in presence or in absence of the doctor. Data are shown as means±SEM. Asterisks (*P<0.04, **P<0.01) refer to the level of statistical significance between values obtained during unattended or attended BP measurement and baseline values.

In the whole group of patients, the average office SBP/DBP values obtained during the visits preceding the study were 153.2±3.4/101.7±2.7 mm Hg. As illustrated in Figure 2, left and right, finger SBP, finger DBP, and HR were stable and superimposable during the baseline period preceding the unattended or attended BP measurements, either when they were recorded together with MSNA (left) or when they were recorded together with SSNA (right). Compared with baseline values, however, during attended BP measurement finger SBP, finger DBP, and HR showed a progressive increase that started approximately by the time of the initiation of automatic BP measurements whereas during unattended BP measurement they showed, again at the time of the beginning of the automatic BP measurements, a slight progressive reduction that plateaued in the final part of the unattended BP measurement period. As shown in Figure 3, left and right, these discrepant hemodynamic changes were accompanied by different changes in sympathetic nerve traffic. Compared with the baseline values (also stable and superimposable between the two BP measurement procedures), during attended BP measurement, SSNA exhibited a progressive increase and a final plateau, whereas during unattended BP measurement, it exhibited an almost specular reduction. In contrast, MSNA exhibited a clearcut reduction during attended BP measurement and a slight progressive increase during the unattended one. Compared with the average of the baseline periods with MSNA or SSNA recording during unattended BP measurement the peak SBP, DBP, HR, and SSNA reductions were smaller and, except for SBP and SSNA, never statistically significant, this being the case also for the peak MSNA increase (Table 1).

**Table 1.**
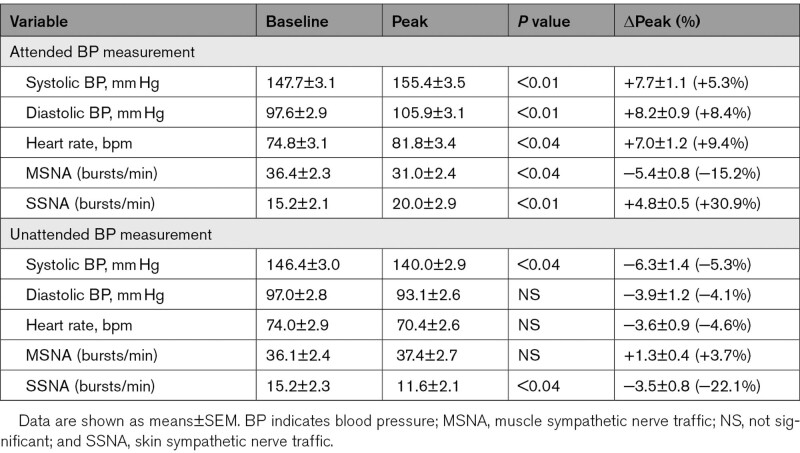
Baseline BP, Heart Rate, and Sympathetic Nerve Traffic Values and Their Peak Responses During Attend and Unattended BP Measurement

The relationships between the peak changes of the different variables during attended or unattended BP measurements are shown in Table 2. During attended BP measurement, the SBP, DBP, and HR increases were significantly related to each other as well as to the SSNA increase or to the MSNA reduction. During unattended BP measurement, the peak BP decrease was significantly related to the concomitant SSNA and HR reductions but not to the MSNA changes. Similar relationships were found when correlation analyses between changes in BP from baseline and peak changes of the various above-mentioned hemodynamic and sympathetic variables were performed.

**Table 2.**
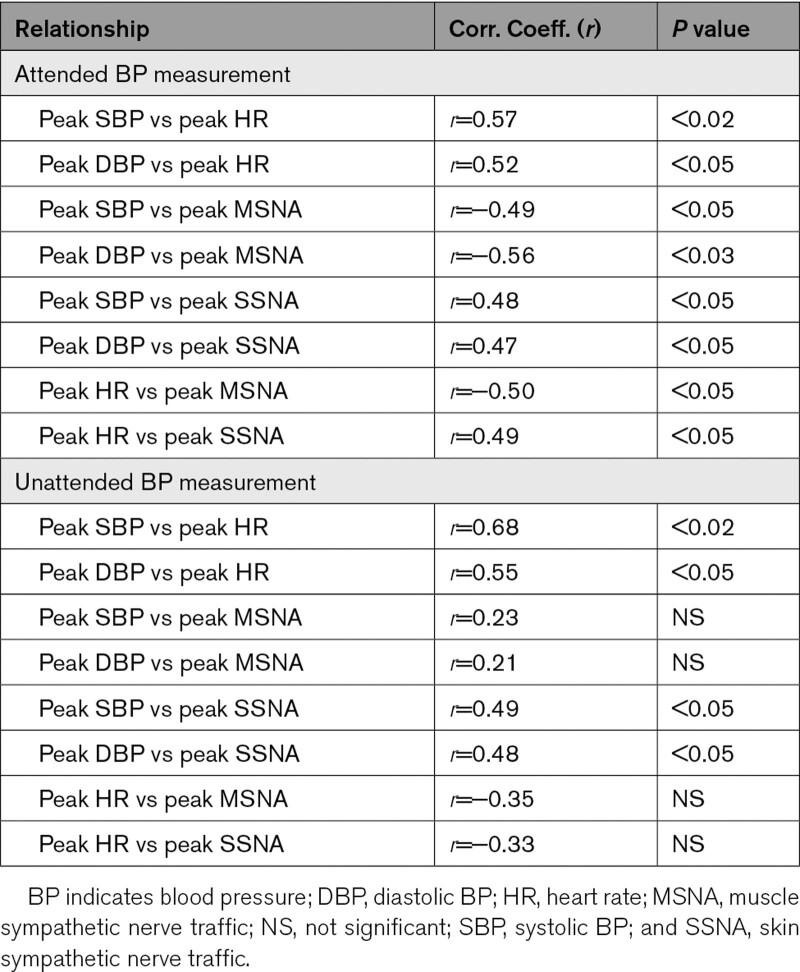
Correlation Coefficients and Related *P* Values of the Relationships Between Peak SBP, DBP, HR, SSNA, and MSNA Changes During Attended or Unattended BP Measurements

## Discussion

In line with previous studies,^11,12^ in our patients, automatic assessment of BP values in presence of a doctor (referred to as attended BP measurement) was associated with an increase in SBP and DBP, a tachycardia, a marked increase in sympathetic nerve traffic to the skin vascular district and a reduction in sympathetic nerve traffic to the skeletal muscle circulation. The novel finding of our study, however, is that this cardiovascular and neural response pattern was strikingly different from the one seen when BP was measured in absence of a doctor (ie, when BP measurements were obtained automatically with the patient alone in the room), which was accompanied by a modest but significant SBP reduction, a decrease in SSNA, and a nonsignificant decrease or no change in HR and MSNA, respectively. Thus, the attended and unattended BP measurement procedures are by no means superimposable for their effect on the cardiovascular system and postganglionic sympathetic nerve activity. That is, attended BP measurement triggers excitatory cardiovascular responses and changes in sympathetic activity that are similar to those that characterize in animals the fight or flight reaction, originally described by Cannon^16^ and confirmed later by other investigators,^17,18^ and in humans the involvement in emotional tasks.^19,20^ This is entirely absent during unattended BP measurement which in contrast appears to be associated with inhibitory effects on both the cardiovascular and the sympathetic nervous system, albeit of less magnitude or consistency than the excitatory effects associated with attended BP measurement. Of interest is the observation that the above-mentioned beat-to beat BP changes appear to be more related in their temporal occurrence to the start of the automated BP measurement rather than to the presence/absence of the doctor.

In our patients, the SBP, DBP, HR, and sympathetic responses to attended BP measurement were less pronounced than those described in previous studies in which these variables were continuously recorded before and during BP measurement by a doctor.^3,11,12^ This may be explained by the fact that, at variance from previous studies, in the present study (1) the doctor was not actively involved in the BP measurements, which were obtained by an automatic device and (2) the attended BP measurement session included a relatively long baseline period. This may have attenuated the emotional impact of the doctor’s presence and favored more modest excitatory cardiovascular responses. However, these attenuated excitatory effects did not make the difference with the effects of the unattended BP measurement procedure negligible because during unattended BP measurement peak SBP was 14.1 mm Hg lower, peak HR 10.6 bpm lower, and peak SSNA 8.5 bursts/min lower than the peak values seen during attended BP measurement. As further discussed below, these considerable differences have important implications for the decision of which should be the threshold and target BP values for the treatment of hypertensive patients, the adoption of values derived from the unattended BP measurement procedure favoring lower values.

The increase in SBP, DBP, and HR that occurred during attended BP measurement leaves no doubt about its origin from an alerting response of the patient, justifying the popular definition of this phenomenon as white coat effect. In the present study, this is further supported by the concomitant increase in SSNA, because sympathetic nerve traffic to the skin vascular district has been shown to respond to emotional stimuli,^21,22^ its quantitative correlation with the BP and HR increases indicating its change as part of an integrated response pattern to an emotional stimulus. In this setting, however, the concomitant reduction in MSNA may appear more difficult to be explained because of the widely held opinion that the sympathetic activation that accompanies emotional behaviors is generalized to the entire sympathetic nervous system. We can speculate the MSNA reduction that accompanies attended BP measurement has a baroreflex origin, that is, it reflects an inhibition of sympathetic control of skeletal muscle circulation by the white coat-dependent BP rise and the consequent baroreceptor stimulation.^11,13^ This implies that the baroreflex exerts a skeletal muscle circulation control which can override an opposing central command. Another more likely possibility, however, is that the MSNA reduction is part of the integrated response of the central nervous system to the emotional stimulus because in both the defense reaction of experimental animals and the emotional states of man, sympathetically mediated cardiac excitation and vasoconstriction are accompanied by skeletal muscle vasodilatation.^18–20^ It should be emphasized that both possibilities imply a heterogeneity of sympathetic control of the cardiovascular system, a concept supported by the evidence by Grassi et al,^13^ Esler et al,^23^ and Esler^24^ in contrast with the older notion affirming that sympathetic activation is always generalized.

Several other results of our study deserve to be mentioned. First, in our patients, unattended BP measurement led to a modest but significant SBP and SSNA reductions, which correlated with each other and with a reduction in HR. This suggests that this BP measurement procedure is not devoid of effects on neural cardiovascular control but that it may somewhat paradoxically have a small relaxing effect on the patient. Second, the results of the present study draw attention to the fact that attended and unattended BP measurements not only lead to different BP but also to different HR values. This has a practical implication because, in recent guidelines, HR is indicated as an important cardiovascular risk factor (especially if values are above 80 bpm), and HR measurement is recommended at any visit in which BP is measured.^25^ Third, in our patients, we found a significant correlation between the peak HR and the peak MSNA or SSNA changes observed during attended or unattended BP measurement. This would suggest that HR in this context may represent a reliable adrenergic marker, capable to mirror the sympathetic changes observed during automated BP measurement. It should be mentioned, however, that a significant correlation, like the one we detected in the present study in a small number of patients, can hardly modify the conclusion of our previous studies showing the limited value of HR as adrenergic marker.^26,27^ Finally, both during attended and unattended BP measurements, the neural and cardiovascular changes occurred during the period in which BP was repeatedly measured by the automatic device. This observation suggests that the reported changes were triggered not just by the presence or absence of the doctor per se but by the different psychological effects of BP measurements. Further data, however, are needed to clarify this point.

Our results have some limitations. One limitation is that the complexity of the experimental procedure restricted the number of patients included in the study. A second limitation is that our findings refer to mild-to-moderate hypertensive patients and thus any extrapolation to patients with more severe high BP states should be made with caution. A third limitation is that, although in a previous study we have shown that over the short-term the pressor and tachycardic responses to attended BP measurement are reproducible,^28^ no data are available on the reproducibility of the cardiovascular responses to attended BP measurement or of the sympathetic nerve traffic responses to either measurement procedure. Finally, in our attended BP measurement session, the doctor was not actively involved in the BP measurements which were obtained, as in the unattended BP measurement session, via an automatic device. In other words, there was no active participation of the doctor at the BP measurement at variance with what may happen in clinical practice. However, doctors make use of automatic BP measurement devices rather than the traditional sphygmomanometer more and more frequently.

## Perspectives

In conclusion, by providing direct evidence that during unattended BP measurement patients’ BP, HR, and skin sympathetic activity exhibits a fall rather than the clearcut rise that accompanies the alerting response elicited by attended BP measurement, our study strongly supports the conclusion that the BP values provided by the two measurement procedures differ and that the difference is far from being quantitatively marginal. This will translate into nonmarginal differences in the medical decision about the threshold and target BP values to consider for antihypertensive treatment that may be only generated by use of different BP measuring procedures rather than by the effects of treatment on cardiovascular events. In this context, however, it should also be mentioned that our observations were collected in untreated hypertensive patients. It would thus be important to investigate whether and to what extent use of antihypertensive drugs directly or indirectly acting on the sympathetic nervous system (beta-blockers, angiotensin-converting enzyme inhibitors, angiotensin II receptor blockers, central agents),^29^ will interfere with the neurogenic and hemodynamic responses to unattended and attended BP measurement and modify the differences described in the present study. Even for central agents, the answer to this question is not obvious, given the evidence that rilmenidine may leave unaffected the BP and sympathetic response to mental stress.^30^

## Sources of Funding

None.

## Disclosures

None.

## Novelty and Significance

### What Is New?

- The present investigation was aimed at providing information on the neurogenic mechanisms underlying the blood pressure (BP) changes detected during BP measurements unattended or attended by the doctor.
- This was done by evaluating in 18 untreated mild-to-moderate essential hypertensive patients the muscle sympathetic nerve traffic (MSNA), skin sympathetic nerve traffic (SSNA) and heart rate (HR) changes during unattended and attended BP measurement.

### What Is Relevant?

- The BP decrease detected during unattended measurement was associated with a reduction in HR and SSNA and almost unchanged MSNA. Conversely, during attended BP measurement, there was an increase in BP, a slight HR and SSNA elevation, and a slight MSNA decrease.
- The peak BP decrease observed during unattended BP measurement was significantly directly related to the concomitant SSNA and HR reductions but not to MSNA changes.
- The peak BP increase observed during attended BP measurement was significantly related to HR elevation, SSNA increase, and to MSNA reduction.
- During unattended BP measurement peak systolic BP was 14.1 mm Hg lower, peak HR 10.6 bpm lower, and peak SSNA 8.5 bursts/min lower than the peak values seen during attended BP measurement.

### Summary

The present study provides for the first time documented evidence that sympathetic mechanisms underpin the BP changes associated with unattended or attended BP measurement. The study findings also show, however, that these sympathetic alterations are etherogeneous in nature, combining excitation in one cardiovascular district with inhibition in another.
